# The Role of Chitinase-3-like Protein-1 (YKL40) in the Therapy of Cancer and Other Chronic-Inflammation-Related Diseases

**DOI:** 10.3390/ph17030307

**Published:** 2024-02-27

**Authors:** Ming-Cheng Chang, Chun-Tang Chen, Ping-Fang Chiang, Ying-Cheng Chiang

**Affiliations:** 1 Department of Isotope Research Application, National Atomic Research Institute, P.O. Box 3-27, Longtan, Taoyuan 325, Taiwan; mcchang@nari.org.tw (M.-C.C.); ctchen@nari.gov.tw (C.-T.C.); ckdopamine@nari.gov.tw (P.-F.C.); 2 Department of Obstetrics and Gynecology, College of Medicine, National Taiwan University, Taipei 100226, Taiwan; 3 Department of Obstetrics and Gynecology, National Taiwan University Hospital, Taipei 100226, Taiwan

**Keywords:** *CHI3L1*(YKL40), targeted therapy, medical diseases, cancers

## Abstract

Chitinase-3-like protein-1 (*CHI3L1*), also known as YKL40, is a glycoprotein that belongs to the chitinase protein family. It is involved in various biological functions, including cell proliferation and tissue remodeling, with inflammatory and immunomodulatory capabilities. Several studies have shown that *CHI3L1*(YKL40) is upregulated in various diseases, such as cancer, asthma, and inflammatory bowel disease, among others. Although the expression level of *CHI3L1*(YKL40) is associated with disease activity, severity, and prognosis, its potential as a therapeutic target is still under investigation. In this review, we summarize the biological functions, pathological roles, and potential clinical applications of specific inhibitors and targeted therapies related to *CHI3L1*(YKL40).

## 1. Introduction

Chitinase-3-like protein-1 (*CHI3L1*), a type of glycoprotein located on chromosome 1q32.1, spans approximately 10 kilobases and consists of 10 exons encoding a 40 kDa glycoprotein that was initially discovered in the mid-1990s. Analyses of *CHI3L1*’s genomic structure have revealed several genetic variants, including single-nucleotide polymorphisms (SNPs) and copy number variations (CNVs) [1,2]. *CHI3L1* is commonly found in both prokaryotes and eukaryotes, and it is known as YKL40 in humans and breast regression protein 39 (BRP-39) in mice [3]. The YKL40 protein, also known as acidic mammalian chitinase-related protein (AMCase), is a type of cytokine that is encoded by *CHI3L1* and is commonly found in various cells and tissues, including immune cells, liver, brain, and tumor tissues [3,4,5,6]. *CHI3L1* is involved in various physiological processes, such as cell proliferation, differentiation, apoptosis, angiogenesis, inflammation, and tissue remodeling [3,4,5]. *CHI3L1*(YKL40) can inhibit oxidative damage in the lungs, enhance adaptive immune responses, regulate cell apoptosis, stimulate macrophage activation, and aid in fibrosis and wound healing [1,6,7].

*CHI3L1*(YKL40) also exhibits a pro-angiogenic effect, which can promote tumor angiogenesis through vascular endothelial cells and maintain vascular integrity through smooth muscle cells [8,9]. Patients with various cancers, including breast, gastrointestinal, liver, prostate, brain, endometrial, and lung cancers, as well as astrocytoma and glioblastoma, have elevated levels of *CHI3L1*(YKL40) in their serum [3,10,11,12]. Several studies have indicated that serum or plasma *CHI3L1*(YKL40) can be used as an indicator of treatment response and prognosis in cancer patients [13,14,15].

*CHI3L1*-mediated signaling transduction pathways, such as MAPK and NF-κB, ERK1/2 and AKT, Wnt/β-catenin, and β-catenin, as well as FAK and STAT3, JNK, and p38, have also been reported in various independent investigations. These pathways indicate that *CHI3L1* could play a role in many different functions across various cell types. Despite *CHI3L1*(YKL40) being extensively studied in immune responses in numerous investigations, the mechanisms by which *CHI3L1*(YKL40) influences the host oncogenic progress are still unclear. In this review, we focus on the role of *CHI3L1*(YKL40) in pathological diseases, including rheumatoid arthritis, neurological and autoimmune diseases, and cancers. We also provide information on the current development of pharmaceuticals targeting *CHI3L1*(YKL40) to elucidate the possible clinical applications of *CHI3L1*(YKL40)-targeted drugs in patients with various diseases.

## 2. Relevant Sections and Discussion

### 2.1. Biology of CHI3L1(YKL40)

YKL40 is a highly conserved chitin-binding glycoprotein that binds heparin and chitin without chitinase activity, and it is mainly located in the extracellular space. The significance of *CHI3L1* in health and disease sets the stage for a comprehensive investigation into its biological functions. One of the key aspects of *CHI3L1* is its involvement in inflammatory responses. *CHI3L1* has been shown to induce the activation of various immune cells, including macrophages and neutrophils. These cells play crucial roles in the inflammatory response. Moreover, *CHI3L1* may upregulate the expression of pro-inflammatory cytokines, such as interleukin-6 (IL-6) and tumor necrosis factor-alpha (TNF-α). It may also be involved in the regulation of anti-inflammatory cytokines, contributing to the balance between pro-inflammatory and anti-inflammatory signals. *CHI3L1* may influence extracellular matrix (ECM) components and tissue-remodeling enzymes as well, contributing to the resolution of inflammation. It may interact with various cell surface receptors, including interleukin-13 receptor alpha 2 (IL-13Rα2) and chondroitin sulfate proteoglycans. These interactions can mediate downstream signaling pathways involved in inflammation. The molecular mechanisms by which *CHI3L1* modulates inflammation have been comprehensively investigated, exploring its interactions with immune cells, cytokines, and signaling pathways. Understanding these intricate relationships provides crucial insights into the regulatory role of *CHI3L1* in maintaining immune homeostasis.

With the exception of immune regulation, *CHI3L1* has been associated with regulating ECM components. It may influence the synthesis, degradation, and remodeling of ECM proteins, such as collagen, fibronectin, and proteoglycans. These proteins may downregulate biological processes, including cell–ECM adhesion, migration, and tissue architecture. *CHI3L1* has also been demonstrated to stimulate fibroblast activation and proliferation. The activation of fibroblasts by *CHI3L1* may lead to the increased production of ECM proteins, promoting tissue remodeling and repair. The alternation of these biological functions may depend on MAPK and PI3K/Akt activation. Furthermore, *CHI3L1* has been associated with the promotion of angiogenesis and the formation of new blood vessels. By influencing endothelial cell function and angiogenic signaling pathways, *CHI3L1* may contribute to the vascularization of tissues during repair processes.

*CHI3L1*’s role also extends to safeguarding against pathogens, mediating responses to antigen-induced and oxidant-induced injuries, regulating inflammation, and orchestrating tissue repair and remodeling. The protein exerts control over essential biological processes, including oxidant injury, apoptosis, pyroptosis, inflammasome activation, Th1/Th2 inflammatory balance, M2 macrophage differentiation, dendritic cell (DC) accumulation, TGF-β1 expression, ECM regulation, and parenchymal scarring [5,6,7]. *CHI3L1* also exerts a chemotactic influence on vascular endothelium and smooth muscle cells. Its regulatory role extends to the modulation of vascular endothelial cell morphology through the stimulation of endothelial tubulogenesis, as well as the facilitation of vascular smooth muscle cell migration and adhesion.

Various investigations have revealed its involvement in a wide range of immune-mediated physiological and pathological processes, some of which include immune regulation, cancer, chronic obstructive pulmonary disease [8,9], and asthma [9,10], indicating that *CHI3L1*(YKL40) may serve as a potential marker of activated macrophages.

### 2.2. Molecular Structure of CHI3L1(YKL40)

**Figure 1:**
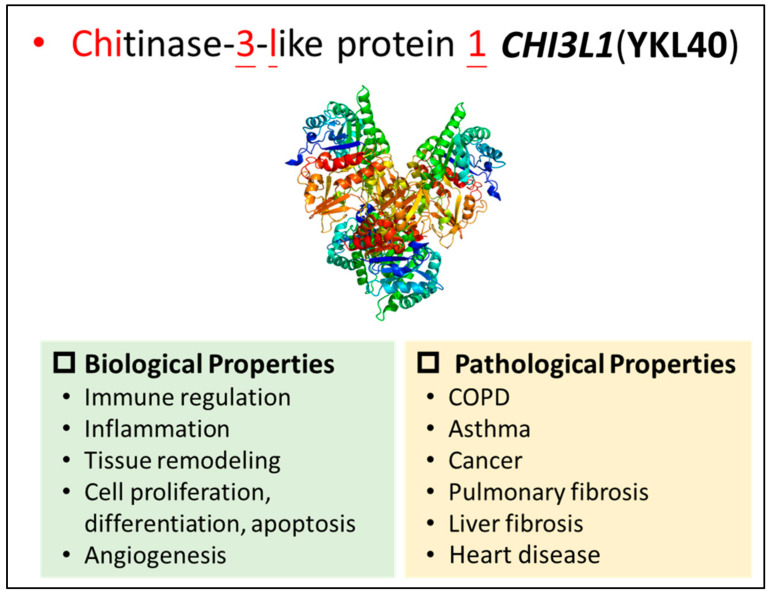
The properties of *CHI3L1*(YKL40). The biological properties include immune regulation, inflammation, tissue modeling, cell proliferation, differentiation, apoptosis, and angiogenesis. The dysregulation of *CHI3L1* is noted in pathological conditions, including COPD, asthma, cancer, pulmonary fibrosis, liver fibrosis, and heart disease.

The native structure of YKL40 has been resolved. Its structure comprises four YKL40 monomers spanning residues 22–383, alongside 117 water molecules. The four crystallographically independent molecules exhibit substantial similarity. In the crystal structure of YKL40, the absence of the initial 21 N-terminal residues forming the leader sequence is notable. The crystal structure of YKL40 also reveals a barrel-shaped glycoside hydrolase family 18 (GH18) domain characteristic of chitinases and chitinase-like proteins [16,17]. The protein also contains several conserved cysteine residues that form disulfide bonds, which are thought to be important for its stability and biological function [11,12]. Three cis peptide bonds are discernible, with particular emphasis on the observation that two of these bonds are situated in the sugar-binding groove. YKL40 is N-glycosylated at Asn60, with two β (1, 4)-linked GlcNAc residues distinctly evident in the electron density. A meticulous examination of the electron density map exposes a sequence mismatch at position 311, where an isoleucine residue is present instead of the anticipated threonine, as indicated by the deposited amino acid sequence in Swiss-Prot entry P36222. This sequence discrepancy is consistent across all four YKL40 molecules in the asymmetric unit, being substantiated through mass spectrometry analysis. The identification of genetic variants of *CHI3L1* and the crystal structure of YKL40 have provided valuable insights into its biological functions and potential therapeutic applications [11,12] (Figure 1).

### 2.3. Tissue Distribution of CHI3L1(YKL40)

YKL40 expression can be context-specific and influenced by factors such as inflammation and tissue remodeling. YKL40 is commonly expressed in connective tissues, including cartilage, tendons, and ligaments. Its presence in these tissues suggests a role in extracellular matrix maintenance and remodeling. Elevated levels of YKL40 are also observed in lung tissues, particularly in conditions associated with inflammation and respiratory diseases. It is often used as a biomarker for assessing airway inflammation and predicting disease severity. In the central nervous system, YKL40 expression can be detected in the brain. It has been shown to be implicated in neuroinflammation and is associated with conditions such as Alzheimer’s disease, in which its levels may be elevated in response to neuronal injury. Moreover, YKL40 is expressed in the liver. Its levels can increase in conditions such as liver fibrosis and cirrhosis, potentially serving as a marker of hepatic inflammation and injury. Finally, YKL40 expression can also be detected in adipose tissue, and its levels may be associated with obesity-related inflammation. The protein’s role in metabolic processes and adipose tissue homeostasis is an area of ongoing research. Understanding the distribution of YKL40 across different tissues provides insights into its diverse functions, ranging from tissue homeostasis to its involvement in inflammatory responses and various diseases.

### 2.4. Physiological Processes Involving CHI3L1(YKL40)

Several investigations have focused on *CHI3L1*-mediated physiological processes. For cellular differentiation and development, *CHI3L1* has been identified as playing an important role in embryogenesis and tissue formation. The protein’s involvement in ECM remodeling and angiogenesis contributes to the restoration of tissue integrity after injury. *CHI3L1* is also associated with wound healing and tissue repair processes. It may promote the migration and activation of cells involved in tissue regeneration, such as fibroblasts and endothelial cells. *CHI3L1* promotes the formation of new blood vessels. This physiological process is crucial for supplying oxygen and nutrients to growing tissues and facilitating their proper development. The role of *CHI3L1* in angiogenesis may involve interactions with endothelial cells and the modulation of signaling pathways associated with blood vessel formation. *CHI3L1* also participates in physiological immune responses and inflammation. It can influence the activation and recruitment of immune cells, contributing to the defense against infections and maintaining immune homeostasis. Moreover, by eliminating local immune responses via *CHI3L1* expression during pregnancy, it has been shown to be implicated in preventing fetal rejection and aiding in processes that facilitate fetal development.

### 2.5. Binding Partners and Signaling Pathways Involving CHI3L1(YKL40)

**Figure 2:**
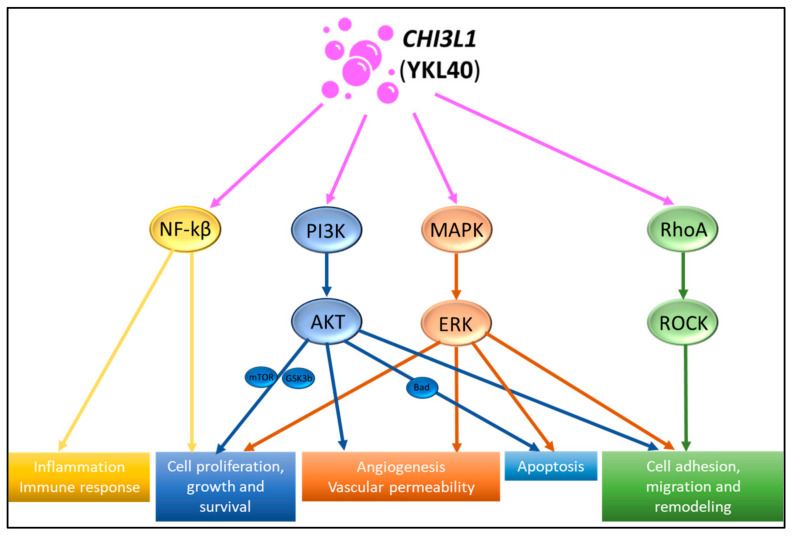
Summary of *CHI3L1*-regulated signal transduction pathways. The signal transduction pathways in relation to *CHI3L1* include the PI3K/AKT, MAPK/ERK, NF-kβ, and RhoA/ROCK pathways.

Several signaling transduction pathways enhanced by *CHI3L1*(YKL40) have been demonstrated to stimulate biological and/or pathological responses. These signal transduction pathways are illustrated in Figure 2.

#### 2.5.1. PI3K/Akt Signaling Pathway

PI3K/Akt is an important signal transduction pathway upregulated by *CHI3L1*(YKL40) to stimulate downstream responses. *CHI3L1*(YKL40) binds to specific receptors on the cell membrane to stimulate PI3K activation and Akt phosphorylation [13,14]. Activated Akt phosphorylates and regulates multiple downstream effectors, including mammalian target of rapamycin (mTOR) [15], glycogen synthase kinase 3β (GSK-3β) [18], and Bcl-2-associated agonist of cell death (Bad) [19]. Akt activation enables it to phosphorylate various downstream targets involved in cell cycle progression, proliferation, metabolism, angiogenesis, and migration, as well as inhibiting apoptosis. Akt activation promotes cell cycle progression by inhibiting GSK-3β, leading to the stabilization of cyclin D1 [16]. Additionally, Akt activation inhibits apoptosis by phosphorylating and inactivating pro-apoptotic proteins such as Bad.

#### 2.5.2. MAPK/ERK Signaling Pathway

The MAPK/ERK signaling pathway involves a series of protein kinases, including mitogen-activated protein kinase (MAPKK or MEK) and extracellular-signal-regulated kinase (ERK) [17,20,21]. *CHI3L1*(YKL40)-mediated signaling activates MAPKK, which, in turn, phosphorylates and activates ERK, and this phosphorylation cascade leads to the activation of ERK, allowing it to translocate to the nucleus and phosphorylate a variety of specific target proteins, including transcription factors and other kinases. These target proteins are involved in regulating gene expression, cell proliferation, and angiogenesis, as well as cell adhesion and migration [22]. ERK activation regulates the expression of genes involved in promoting cell cycle progression and cell growth, thereby contributing to increased cell proliferation. Additionally, ERK activation can inhibit apoptosis by modulating the expression of anti-apoptotic proteins and inhibiting pro-apoptotic signals [13].

#### 2.5.3. NF-κB Signaling Pathway

*CHI3L1*(YKL40) can regulate inflammatory responses by activating the NF-κB signaling pathway [22]. One of the main regulators in the NF-κB pathway is IκB, which sequesters NF-κB proteins in an inactive state in the cytoplasm. In this pathway, *CHI3L1*(YKL40) promotes the degradation of IκBα (inhibitor of κB), which leads to the release and translocation of NF-κB proteins from the cytoplasm to the nucleus. NF-κB proteins act as transcription factors, binding to specific DNA sequences and modulating the expression of pro-inflammatory cytokines, chemokines, and adhesion molecules that contribute to the initiation and amplification of inflammatory responses [23,24]. Additionally, NF-κB activation by *CHI3L1*(YKL40) may also influence immune regulation by modulating the expression of immune-related genes involved in inflammation, immune responses, and cell survival [22,23,24].

#### 2.5.4. RhoA/ROCK Signaling Pathway

The RhoA/ROCK pathway is a well-known signaling cascade involved in various cellular processes, such as cell migration, adhesion, and cytoskeletal rearrangement [25,26]. In this pathway, *CHI3L1*(YKL40) activates RhoA, thereby influencing cell cytoskeletal reorganization and migration [27]. Initially, *CHI3L1*(YKL40) interacts with specific cell surface receptors, such as receptor for advanced glycation end products (RAGE) and IL-13 receptor α2 (IL-13Rα2), to stimulate RhoA activation, leading to the exchange of GDP for GTP on RhoA [17,28]. Moreover, activated RhoA interacts with ROCK, promoting its activation. ROCK phosphorylates various substrates, including myosin light chain (MLC) and LIM kinase (LIMK), which results in cytoskeletal reorganization and the modulation of cell motility and adhesion [29]. This can contribute to immune cell recruitment and tissue inflammation. In addition, *CHI3L1*(YKL40)-mediated RhoA/ROCK activation has been shown to be implicated in tissue-remodeling processes, such as extracellular matrix deposition, fibrosis, and wound healing. RhoA/ROCK signaling influences the contractility of fibroblasts and myofibroblasts, resulting in tissue restructuring and repair.

## 3. Pathological Roles of *CHI3L1*(YKL40)

### 3.1. CHI3L1(YKL40) in Inflammation-Related Diseases

Chronic obstructive pulmonary disease (COPD) is a progressive lung disorder characterized by a persistent airflow limitation and chronic inflammation. Previous studies investigating the correlation between *CHI3L1*(YKL40) expression levels and COPD progression have reported elevated levels of *CHI3L1*(YKL40) in the blood, sputum, and lung tissue of individuals with COPD compared to healthy individuals or those with other respiratory conditions [30,31,32,33,34]. Moreover, Matsuura et al. also reported that higher *CHI3L1*(YKL40) levels were associated with more severe COPD phenotypes, including a decline in lung function and emphysema severity, and an increased risk of acute exacerbations [35]. Chronic inflammation plays a crucial role in the disease pathogenesis of COPD, and elevated *CHI3L1*(YKL40) levels may contribute to the perpetuation of inflammation and tissue-remodeling processes in COPD [9,10]. Furthermore, several studies have found a positive correlation between *CHI3L1*(YKL40) expression and reduced lung function, as measured by the decreased forced expiratory volume in one second (FEV1) [36] and forced vital capacity (FVC) [37]. These findings suggest that *CHI3L1*(YKL40) may be involved in the development and progression of airflow limitation in COPD. Due to its strong association with disease severity, the feasibility of *CHI3L1*(YKL40) as a potential biomarker for COPD has been evaluated. Measuring *CHI3L1*(YKL40) expression levels could aid in the diagnosis and assessment of disease severity, as well as the prediction of outcomes in COPD patients. While the correlation between *CHI3L1*(YKL40) expression and COPD has been observed, further research is needed to elucidate the underlying mechanisms of *CHI3L1*(YKL40) in COPD pathogenesis and its potential as a therapeutic target for this complex lung disease.

Asthma is a chronic respiratory disease characterized by airway hypersensitivity and narrowing. Previous studies have found that *CHI3L1*(YKL40) expression levels are significantly elevated in asthma patients, and it may play an important role in the pathogenesis of asthma [30,38,39]. A positive correlation between *CHI3L1*(YKL40) expression levels, the degree of airflow limitation, and the severity of symptoms has been noted in asthma patients [38]. Additionally, elevated levels of *CHI3L1*(YKL40) have also been associated with an increased risk of frequent acute exacerbations, the poor control of airway symptoms, and the clinical prognosis of asthma [30,39,40]. These findings suggest that *CHI3L1*(YKL40) may serve as a potential prognostic marker to assess disease severity and predict the risk of complications in asthma patients [41]. However, the underlying mechanism of *CHI3L1*(YKL40) in asthma is not fully understood; thus, it is necessary to understand the complex roles of *CHI3L1*(YKL40) in asthma pathogenesis and its potential as a target for treatment.

YKL40, the protein product of *CHI3L1*, is an inflammatory glycoprotein produced by various cell types, including cancer, immune, and connective tissue cells [5,42,43]. The possible mechanisms by which immune responses are regulated by *CHI3L1*(YKL40) have been also investigated. It can enhance T-cell responses and activate immune cells, such as macrophages and dendritic cells [42,44]. It can also promote immune responses by increasing the production of inflammatory cells, chemokines, and cytokines, such as IL-13, to induce cell apoptosis [3,17]. *CHI3L1*(YKL40) exhibits specific regulatory effects in some fibrotic diseases such as pulmonary fibrosis [6], liver cirrhosis [45], and heart disease [46,47]. It can increase fibroblast proliferation [42] and collagen synthesis [48], inhibit oxidative damage in the lungs, enhance adaptive immune responses, regulate cell apoptosis, stimulate macrophage activation, and contribute to fibrosis and wound healing [49,50].

### 3.2. Oncogenic Roles of CHI3L1(YKL40)

*CHI3L1*(YKL40) plays a role in tumorigenesis, including participation in cell proliferation, differentiation, apoptosis, angiogenesis, inflammation, and tissue remodeling [5,6,51]. With regard to cancer angiogenesis, *CHI3L1*(YKL40) facilitates tumor vascularization through endothelial cells and maintains vascular integrity through smooth muscle cells [52,53]. Several studies have reported a strong correlation between elevated expression levels of *CHI3L1* (YKL40) in the serum of patients with various cancers, including breast, gastrointestinal, liver, prostate, brain, endometrial, and lung cancers, as well as astrocytomas [5,51,54]. Higher expression levels of *CHI3L1*(YKL40) have been associated with advanced stages of cancer, increased tumor size, and poor prognosis [6,55,56]. Several studies have indicated that serum or plasma levels of *CHI3L1*(YKL40) can be used to assess treatment response and prognosis in ovarian cancer patients, in which a higher expression of *CHI3L1*(YKL40) was associated with poorer outcomes [55,57]. Translational experiments have revealed that *CHI3L1*(YKL40) promotes the expression of the Mcl-1 protein, inhibiting the apoptotic effect induced by paclitaxel in ovarian cancer cells and conferring resistance to paclitaxel [55]. Additionally, *CHI3L1*(YKL40) has a significant impact on the generation of ovarian cancer stem cells by activating the Akt and ERK signaling pathways to promote the expression of β-catenin and SOX2, thus endowing ovarian cancer cells with characteristics of cancer stem cells [57]. Overall, *CHI3L1*(YKL40) plays a significant role in cancer development and progression. However, whether *CHI3L1*(YKL40) can further serve as a therapeutic target for tumors requires further research.

## 4. *CHI3L1*(YKL40)—Related Therapy

Due to its role as a biomarker in some medical diseases and cancers, several studies investigated its potential as a therapeutic target in various diseases. Some studies have been conducted or are currently ongoing to investigate the effects of therapy targeting *CHI3L1*(YKL40) in the treatment of various diseases. In the following table, we summarize the status of these studies (Table 1).

### 4.1. Rheumatoid Arthritis

Rheumatoid arthritis (RA) is an autoimmune disease characterized by chronic inflammation of the joints. It is believed that *CHI3L1*(YKL40) is implicated in the pathogenesis of RA and contributes to the inflammatory process and joint damage observed in this disease [58,59,60]. Targeting *CHI3L1*(YKL40) has been considered a potential therapeutic strategy for modulating the immune response and reducing inflammation in RA. A monoclonal antibody (mAb) targeting *CHI3L1*(YKL40) was developed to neutralize its activity or block its interaction with cellular receptors [61]. These antibodies can potentially inhibit the pro-inflammatory effects of *CHI3L1*(YKL40), such as cytokine production and immune cell activation. By reducing inflammation, these therapies aim to alleviate the symptoms and slow down joint damage in RA. Moreover, investigations have also indicated small molecules can be designed to inhibit the enzymatic activity of *CHI3L1*(YKL40). This approach focuses on blocking the chitinase activity of *CHI3L1*(YKL40), which has been shown to be implicated in tissue remodeling and inflammatory responses. Gene silencing techniques, including RNA interference (RNAi), specifically targeting and reducing *CHI3L1*(YKL40) expression in affected joints have also been explored. It is important to mention that, while these approaches show promise, they are still in the early stages of development, and further research is required to validate the efficacy and safety of treating RA. Clinical trials are necessary to assess the therapeutic potential of *CHI3L1*(YKL40)-targeted therapies, including the effects on disease activity, symptoms, and joint damage in RA patients.

### 4.2. Asthma

The level of *CHI3L1*(YKL40) is correlated with disease severity in asthma patients. It is produced by various cell types, including airway epithelial cells, macrophages, and fibroblasts, and it has been shown to be implicated in the pathogenesis of asthma. Elevated levels of *CHI3L1*(YKL40) in blood or sputum samples could potentially aid in identifying patients with a higher risk of severe asthma attacks, as well as guiding treatment decisions [41,62]. Various studies have explored the potential of blocking *CHI3L1*(YKL40) activity or neutralizing its effects to reduce airway inflammation and improve asthma symptoms [63]. Other in vivo studies found that the mAb was well-tolerated and led to significant improvements in lung function and asthma symptoms [64].

### 4.3. Osteoarthritis

*CHI3L1*(YKL40) has been found to be elevated in the synovial fluid and serum of osteoarthritis patients, suggesting its involvement in the disease process, and the use of *CHI3L1*(YKL40) as a target for therapy in osteoarthritis is a topic of ongoing research [65,76]. Some studies have investigated the potential of natural compounds or plant extracts in inhibiting *CHI3L1*(YKL40) activity. Another strategy involves using antibodies to neutralize the effects of *CHI3L1*(YKL40) in order to reduce cartilage damage, alleviate joint pain, and preserve various functions [66]. By binding to *CHI3L1*(YKL40) and preventing its interaction with other molecules or receptors, this approach aims to mitigate the pathological processes associated with osteoarthritis.

### 4.4. Alzheimer’s Disease

Previous investigations have demonstrated that *CHI3L1*(YKL40) is involved in inflammatory processes and neurodegenerative disorders, such as Alzheimer’s disease [77,78]. Elevated levels of *CHI3L1*(YKL40) have been observed in the cerebrospinal fluid (CSF) and brain tissue of individuals with Alzheimer’s disease [78,79]. It has been suggested that *CHI3L1*(YKL40) may contribute to the neuroinflammatory response and the formation of amyloid-beta plaques, which are a hallmark of Alzheimer’s disease [77,78]. Additionally, *CHI3L1*(YKL40) has been associated with tau protein pathology and neurofibrillary tangles, another characteristic feature of the disease. Research on targeting *CHI3L1*(YKL40) or inhibiting its activity as a therapy against Alzheimer’s disease is still ongoing. However, their use in long-term treatment is limited due to their potential side effects. Small molecular drugs targeting *CHI3L1*, K284-6111 (2-({3-[2-(1-cyclohexen-1-yl)ethyl]-6,7-dimethoxy-4-oxo-3,4-dihydro-2-quinazolinyl}ulfanyl)-*N*-(4-ethylphenyl)butanamide), significantly reduced Aβ1–42-induced β-secretase activity and Aβ generation in ex vivo investigations [67,68]. These results suggest that *CHI3L1*(YKL40) inhibitors could be applicable intervention drugs in amyloidogenesis and neuroinflammation. However, such therapeutic strategies have not led to significant improvements in cognitive function.

### 4.5. Multiple Sclerosis

Elevated levels of *CHI3L1*(YKL40) have been observed in the cerebrospinal fluid and blood of multiple sclerosis patients, particularly during active disease phases [69,80]. Several preclinical studies have investigated the effects of *CHI3L1*(YKL40) in experimental models of multiple sclerosis, such as animal models of autoimmune encephalomyelitis (EAE), which mimic certain aspects of human multiple sclerosis [81,82]. These studies observed a potential involvement of *CHI3L1*(YKL40) in disease progression and demonstrated that targeting *CHI3L1*(YKL40) might have therapeutic benefits. Komori et al. found that the level of *CHI3L1*(YKL40) in CSF was significantly reduced in multiple sclerosis patients receiving daclizumab, and the inhibition of *CHI3L1*(YKL40) was correlated with the inhibition of NFL and a lack of disease progression [70]. Whether *CHI3L1*(YKL40) can further serve as a therapeutic target for multiple sclerosis requires further research.

### 4.6. Cancers

Elevated *CHI3L1*(YKL40) expression has been observed in several cancer types, including brain, breast, lung, ovarian, colorectal, and prostate cancers [54,83]. High *CHI3L1*(YKL40) levels are often associated with aggressive tumor behavior, cancer metastasis, and poor prognosis [51]. There are various therapeutic drugs that target *CHI3L1*(YKL40) currently being researched and developed. These drugs act as typical *CHI3L1*(YKL40) inhibitors, suppressing its biological activity and expression, thereby reducing its impact on disease progression. Some known *CHI3L1*(YKL40) inhibitors include GM-CT-01 from GlycoMimetics (NCT01246984) and ONO-7475 from Novartis. These drugs are currently undergoing clinical trials for the treatment of cancer and inflammatory diseases. Additionally, other drug candidates are being studied and developed, which may become *CHI3L1*(YKL40)-targeted therapeutic drugs in the future.

Monoclonal antibodies (mAb) against *CHI3L1*(YKL40) have been developed as potential therapeutic agents that neutralize it or prevent its binding to receptors, thus inhibiting its pro-tumorigenic effects [84]. Antibody-based therapies also facilitate the immune-mediated clearance of *CHI3L1*(YKL40)-expressing cancer cells. In vitro studies have demonstrated that *CHI3L1*(YKL40) inhibition may have an influence on angiogenesis, migration, and immune response in various tumor cells [71,85]. However, the complexity, heterogeneity, and various molecular pathways all contribute to cancer development and the therapeutic response in the tumor microenvironment. *CHI3L1*(YKL40) may play an important role in the complex processes involved in cancer progression; therefore, while modulating *CHI3L1*(YKL40) may not be sufficient to comprehensively treat all cancers, it shows promise against some cancers. Based on a neutralizing mAb, our group demonstrated the use of a radioisotope-conjugated antibody to generate a novel radiopharmaceutical for inhibiting ovarian cancer progression [72]. Our study demonstrated that the radiopharmaceutical significantly inhibited tumor growth in ovarian cancer xenograft mice, which is a promising result [72].

Gene therapies, including siRNAs or short hairpin RNAs (shRNAs), have also been explored for inhibiting *CHI3L1*(YKL40) expression. Several RNA interference (RNAi) sequences (e.g., 5’-GUGGAAGGUGACAGCUCAATT-3’) have been designed and used in various preclinical studies to specifically inhibit the expression of *CHI3L1*(YKL40) [73]. Inhibiting *CHI3L1*(YKL40) production through RNAi has shown potential in impeding cancer cell proliferation and inducing cell death, leading to a decreased tumor size [73].

Combinational therapies utilizing pharmaceuticals targeting *CHI3L1*(YKL40) combined with chemotherapy, radiotherapy, or immunotherapy have also been explored. *CHI3L1*(YKL40) may serve as a new target for anti-angiogenic therapy that can be combined with neoadjuvant chemotherapy to reduce chemoresistance and inhibit metastasis in breast cancer patients [74]. Ku et al. also indicated that a combination strategy involving *CHI3L1*(YKL40) inhibition by shRNA and several anticancer drugs, including cisplatin, etoposide, and doxorubicin, could enhance glioblastoma cell death [75]. *CHI3L1*(YKL40) can regulate immune checkpoint gene expression such as PD-L1, PD-L2, PD-1, LAG3, and TIM3, which may enhance immune suppression in the tumor microenvironment, thus resulting in cancer progression and lymphatic spread. It indicates that targeting *CHI3L1*(YKL40) can augment the efficacy of an immune checkpoint blockade in response to anti-PD-1 therapy in malignancies [86]. However, more preclinical and clinical studies are necessary to assess the safety, efficacy, and long-term effects of *CHI3L1*(YKL40)-targeted therapies in cancer patients. Further investigations of the biological and therapeutic interactions between *CHI3L1*(YKL40)-targeted therapy and chemotherapy, radiotherapy, or immunotherapy are also warranted.

## 5. Conclusions

There are significant correlations between high expression levels of *CHI3L1*(YKL40) and disease severity or prognosis in several malignant and non-malignant diseases. The functions of *CHI3L1*(YKL40) in various physiological conditions include cell proliferation, differentiation, apoptosis, angiogenesis, inflammation, and tissue remodeling. Dysregulation of *CHI3L1*(YKL40) promotes cell proliferation, invasion, migration, tumor angiogenesis, carcinogenesis, and chemoresistance. Therefore, *CHI3L1*(YKL40) has the potential to serve as a therapeutic target. *CHI3L1*(YKL40) inhibition can alter signaling transduction and oncogenic processes, attenuate immune checkpoint gene expression, and suppress angiogenic and metastatic properties to inhibit cancer progression. The impacts of *CHI3L1*(YKL40)-targeted therapy on various diseases and cancer treatment strategies remain to be elucidated. The better we understand the effects and mechanisms of *CHI3L1*(YKL40), the more effectively we can apply *CHI3L1*(YKL40)-targeted therapy in clinical practice.

## Figures and Tables

**Table 1:** *CHI3L1*(YKL40)—Related Therapy.

Rheumatoid arthritis	mAb neutralizes *CHI3L1* activity or block its interaction with cellular receptors and alleviate symptoms and slow down joint damage in RA Small molecules inhibit the activity of *CHI3L1*, which has been implicated in tissue remodeling and inflammatory responses Gene silencing technique including RNA interference (RNAi) to specifically target and reduce *CHI3L1* expression, mitigating its pro-inflammatory effects and dampening the immune response in RA	[58,59,60,61]
Asthma	mAb blocks *CHI3L1* activity or neutralizes its effects to reduce airway inflammation and improve asthma symptoms	[62,63,64]
Osteoarthritis	Natural compounds or plant extracts show inhibitory effects on *CHI3L1* expression or activity in experimental models mAb targeting *CHI3L1* prevents its interaction with other molecules or receptors to mitigate the pathological processes associated with osteoarthritis	[65,66]
Alzheimer’s disease	Ibuprofen and aspirin target *CHI3L1* expression or inhibit its activity to reduce inflammation in the brain and potentially slow down the progression of Alzheimer’s disease Small molecular drug targeting *CHI3L1*, K284-6111 (2-({3-[2-(1-cyclohexen-1-yl)ethyl]-6,7-dimethoxy-4-oxo-3,4-dihydro-2-quinazolinyl}ulfanyl)-N-(4-ethylphenyl)butanamide), reduce Aβ1–42-induced β-secretase activity and Aβ generation, but does not improve the cognitive function	[67,68,69]
Multiple sclerosis	Daclizumab significantly reduces *CHI3L1* level in CSF which is correlated with inhibition of NFL and lack of disease progression	[70]
Cancers	*CHI3L1* inhibitors, including GM-CT-01 and ONO-7475, undergo clinical trials for the treatment of cancer mAbs targeting *CHI3L1* neutralize its activity to inhibit its pro-tumorigenic effects Radioisotope-conjugated antibody targeting *CHI3L1* to generate a novel radiopharmaceutical drug to inhibit ovarian cancer progression in xenograft mice Combination with anti-angiogenic therapy targeting *CHI3L1* and neoadjuvant chemotherapy to reduce chemoresistance and inhibit metastasis in breast cancer patients Combination strategy with *CHI3L1* inhibition by shRNA and several anticancer drugs, including cisplatin, etoposide, and doxorubicin could enhance glioblastoma cell death	[71,72,73,74,75]

## Data Availability

Data sharing is not applicable.
